# Postoperative respiratory depression in patients on sublingual buprenorphine: a retrospective cohort study for comparison between postoperative continuation and discontinuation of buprenorphine

**DOI:** 10.1186/s40981-022-00535-2

**Published:** 2022-06-21

**Authors:** Ryu Komatsu, Michael D. Singleton, Katherin A. Peperzak, Jiang Wu, Emily M. Dinges, Laurent A. Bollag

**Affiliations:** 1 Department of Anesthesiology and Pain Medicine, University of Washington, Box 356540, 1959 NE Pacific Street, Seattle, WA 98195 USA; 2 Institute of Translational Health Sciences, University of Washington, Seattle, WA USA

**Keywords:** Buprenorphine, Opioid use disorder, Respiratory depression, Respiratory complications

## Abstract

**Background:**

We tested the hypothesis that patients who continued buprenorphine postoperatively experience postoperative respiratory depression less frequently than those who discontinued buprenorphine.

**Methods:**

This is a retrospective cohort study of patients who were on buprenorphine preoperatively. The primary outcome was postoperative respiratory depression as defined by respiratory rate < 10/minute, oxygen saturation (SpO_2_) < 90%, or requirement of naloxone for 48 h postoperatively. The secondary outcome was the composite of postoperative respiratory complications. The associations between postoperative buprenorphine continuation and respiratory depression and respiratory complications were estimated using separate multivariable logistic regression models, including demographic, intraoperative characteristics, and preoperative buprenorphine dose as covariates.

**Results:**

Postoperative buprenorphine continuation was not associated with postoperative respiratory depression (adjusted odds ratio (OR), 1.11, 95% confidence interval (CI), 0.61 to 1.99, *P*=0.72). In subanalysis stratified by the preoperative buprenorphine dose, buprenorphine continuation was not associated with postoperative respiratory depression either when preoperative buprenorphine dose was high (≥16 mg daily) or low (<16 mg daily). Postoperative buprenorphine continuation was associated with lower incidence of postoperative respiratory complications (adjusted OR, 0.43, 95% CI, 0.21 to 0.86, *P*=0.02).

**Conclusions:**

Continuing buprenorphine was not associated with respiratory depression, but it was associated with a lower incidence of respiratory complications.

**Supplementary Information:**

The online version contains supplementary material available at 10.1186/s40981-022-00535-2.

## Background

Buprenorphine is a partial mu-opioid agonist with a high-receptor binding affinity [1, 2] used for both chronic pain and medication-assisted treatment for opioid use disorder. Within the therapeutic dose range, buprenorphine occupies mu opioid receptors (16 mg blocks > 80% of available mu-receptors) [3] and prevents other full agonists from binding. Postoperative pain management of patients on buprenorphine has been thought to be difficult even with high doses of additional opioid analgesics [4, 5]. For this reason, perioperative discontinuation of buprenorphine has been historically recommended [6, 7].

However, several recent studies reported higher pain scores [8] and a trend towards larger opioid dose requirements in the immediate postoperative period in patients who discontinued, versus continued, buprenorphine perioperatively [9]. Therefore, it is possible that patients who continue to receive buprenorphine postoperatively may experience less pain and require smaller dose of opioids, which results in less respiratory depression.

We therefore tested the hypotheses that patients who continue buprenorphine during the immediate postoperative period experience postoperative respiratory depression and respiratory complications less frequently than those who discontinue.

## Methods

After institutional review board approval and waiver of consent (University of Washington IRB ID: STUDY00012186, approval date: 1/11/2021), we obtained data for adults taking buprenorphine preoperatively who underwent in-patient non-cardiac surgery under general or regional anesthesia. We performed a retrospective cohort analysis restricting our study to patients requiring postoperative hospital admission for at least 48 h at the University of Washington Medical Center (UWMC) or Harborview Medical Center (HMC) in Seattle, Washington between 2013 and 2020. Patients were excluded from the study if during the first 48 h postoperatively they (1) received epidural analgesia containing opioids (epidural opioids were not documented in our electronic medical record), (2) received mechanical ventilation with sedation, or (3) had two or more surgeries. This study followed the Strengthening the Reporting of Observational Studies in Epidemiology (STROBE) reporting guideline.

Patients were identified as taking buprenorphine preoperatively if (1) there were outpatient orders for buprenorphine before the index surgery, (2) there were inpatient orders for buprenorphine that were flagged as home medications before the index surgery through post-operative hospital discharge, or (3) buprenorphine was listed as a medication in the admission note or the pre-anesthesia assessment note.

Surgery types were initially categorized using the Agency for Healthcare Research and Quality (AHRQ) Clinical Classification procedure category and were assigned based on relative value unit (RVU) surgical current procedural terminology (CPT) codes from professional fee billing [10]. This identified specific surgery types, which were subsequently categorized into 13 categories based on the operative body site.

### Outcomes

The primary outcome was postoperative respiratory depression as defined by respiratory rate < 10/minute, oxygen saturation (SpO_2_) < 90%, or requirement of naloxone to reverse the effect of opioids for 48 h postoperatively. The secondary outcome was the composite of postoperative respiratory complications defined as any of the following diagnoses determined by International Classification of Diseases (ICD)-9 and ICD-10 codes during postoperative admission**:** pneumococcal pneumonia; other bacterial pneumonia; bronchopneumonia, organism unspecified; pneumonia, organism unspecified; pulmonary collapse; pulmonary insufficiency following trauma and surgery; acute respiratory failure; other pulmonary insufficiencies, not elsewhere classified; apnea; other respiratory anomalies; respiratory arrest; pneumonia due to *Streptococcus pneumoniae*; pneumonia due to *Hemophilus influenzae*; bacterial pneumonia, not elsewhere classified; pneumonia, unspecified organism; acute respiratory distress syndrome; acute respiratory failure; respiratory failure, unspecified, unspecified whether with hypoxia or hypercapnia; atelectasis; other pulmonary collapses; apnea, not elsewhere classified; and respiratory arrest (Supplemental Table 1).

Additionally, acute pain intensity, defined as the time-weighted average (TWA) pain score, and opioid dose requirements in morphine milligram equivalents (MME) for 48 h postoperatively, were reported. Postoperative pain was evaluated by nurses using an 11-point numeric rating scale (NRS) (0=no pain, 10=worst pain possible) every 4 h until 48 h postoperatively. TWA pain score is equal to the sum of the portion of each time interval in-between two adjacent pain score measurements multiplied by the average of the corresponding two pain scores, and that was then divided by the time interval between the first and the last pain score measurements. Opioid dose requirements in MME for 48 h postoperatively, not including the dose of buprenorphine given postoperatively, was calculated using the Multicenter Perioperative Outcomes Group conversion table [11].

### Statistical analyses

Univariable tests of association between demographic, intraoperative and postoperative characteristics, and postoperative buprenorphine continuation were conducted using Pearson’s chi-squared test for categorical variables and *t* test or Wilcoxon Mann-Whitney *U* test for continuous variables, as appropriate. Distributions of continuous variables were assessed visually using histograms.

The associations between postoperative buprenorphine continuation and respiratory depression and respiratory complications were estimated using separate multivariable logistic regression models. We hypothesized that the associations would be different for patients who were receiving a high (≥16 mg daily) versus low (<16 mg daily) dose of buprenorphine. Therefore, in addition to primary analysis without stratification by preoperative buprenorphine dose, we developed models in which postoperative buprenorphine continuation, preoperative buprenorphine dose (high/low), and their interaction were included as covariates.

A purposeful variable selection strategy, as described by Bursac et al., was utilized for variable selection and model development [12]. Variable entry criteria into the multivariable logistic regression model was *P* < 0.2 for univariable association with postoperative buprenorphine continuation (Table 1), and variable retention criteria in the multivariable model were *P* < 0.1, or variables which changed any of coefficient estimates of the variables of primary interest by > 10%. Calibration and discrimination of the final logistic regression models were assessed using the Hosmer-Lemeshow goodness of fit test and the c-statistic, respectively [13].

**Table 1:** Demographic, preoperative, operative, and postoperative covariates. Summary statistics presented as *N* (%) of patients, mean ± SD, or median [IQR]

	Buprenorphine continued (*n*=121)	Buprenorphine discontinued (*n*=199)	*P* value
Year of surgery, *N* (%)			0.001
2013	2 (1.7)	10 (5.0)	
2014	0 (0)	10 (5.0)	
2015	3 (2.5)	12 (6.0)	
2016	8 (6.6)	19 (9.6)	
2017	13 (10.7)	33 (16.6)	
2018	19 (15.7)	36 (18.1)	
2019	43 (35.5)	34 (17.1)	
2020	33 (27.3)	45 (22.6)	
Hospital, *N* (%)			0.09
HMC	78 (64.5)	146 (73.4)	
UWMC	43 (35.5)	53 (26.6)	
Surgery category by body system, *N* (%)			0.02
Digestive	19 (15.7)	25 (12.6)	
Musculoskeletal	49 (40.5)	111 (55.8)	
Integumental	15 (12.4)	28 (14.1)	
Others^a^	38 (31.4)	35 (17.6)	
Anesthesia			0.16
Regional anesthesia	27 (22.3)	32 (16.1)	
No regional anesthesia	94 (77.7)	167 (83.9)	
Duration of surgery, minutes	147 ± 112	139 ± 116	0.56
Age, years	46 ± 16	45 ± 13	0.53
Sex, *N* (%)			0.09
Male	60 (49.6)	118 (59.3)	
Female	61 (50.4)	81 (40.7)	
Ethnicity			0.94
American Indian or Alaska Native	7 (5.8)	11 (5.5)	
African American	9 (7.4)	20 (10.1)	
White	97 (80.1)	157 (78.9)	
Others	6 (2.0)	8 (4.0)	
Unknown	2 (1.7)	3 (1.5)	
BMI, kg/m^2^	27.0 ± 6.3	28.0 ± 7.7	0.21
ASA physical status, *N* (%)			0.46
1/2	40 (33.1)	77 (38.7)	
3	66 (54.5)	104 (52.3)	
4/5	15 (12.4)	18 (9.0)	
Emergency surgery, *N* (%)	26 (21.5)	46 (23.1)	0.73
Medical history, *N* (%)			
Chronic pulmonary disease, *N* (%)	34 (28.1)	53 (26.6)	0.77
Obstructive sleep apnea, *N* (%)	44 (36.4)	62 (31.2)	0.34
Hypertension, *N* (%)	36 (29.8)	58 (29.2)	0.91
Congestive heart failure, *N* (%)	9 (7.4)	10 (5.0)	0.38
Diabetes mellitus, *N* (%)	12 (9.9)	26 (13.1)	0.40
Tobacco use, *N* (%)	44 (36.4)	66 (33.2)	0.56
Indication of buprenorphine, *N* (%)			0.01
Chronic pain	20 (16.5)	58 (29.1)	
Opioid use disorder	101 (83.5)	141 (70.9)	
Use of preoperative drugs, *N* (%)			
Benzodiazepine	19 (15.7)	41 (20.6)	0.28
Antidepressants	63 (52.1)	78 (39.2)	0.02
Gabapentinoids	42 (34.7)	82 (41.2)	0.25
Muscle relaxants	23 (19.0)	44 (22.1)	0.51
Preoperative daily dose of buprenorphine, mg	12.0 [8.0–16.0]	8.0 [8.0–16.0]	0.06
Preoperative daily buprenorphine dose ≥ 16mg	57 (47.1)	80 (40.2)	0.23
Intraoperative medications, *N* (%)			
Acetaminophen	29 (24.0)	36 (18.1)	0.21
NSAIDs	18 (14.9)	20 (10.1)	0.20
Ketamine	57 (47.1)	90 (45.2)	0.74
Intraoperative opioid dose in MME	75 [45–150]	75 [53–150]	0.86
Postoperative medications (within 48 h after surgery)^b^, *N* (%)			
Acetaminophen	107 (88.4)	186 (93.5)	0.12
NSAIDs	38 (31.4)	62 (31.2)	0.96
Gabapentinoids	70 (57.9)	109 (54.8)	0.59
Ketamine infusion	46 (38.0)	73 (36.7)	0.81
Lidocaine infusion	24 (19.8)	35 (17.6)	0.62

*Abbreviations*: *SD* standard deviation, *IQR* interquartile range, *BMI* body mass index, *ASA* American Society of Anesthesiologists, *MME* morphine milligram equivalents, *NSAIDs* non-steroidal anti-inflammatory drugs

^a^Others include endocrine, cardiovascular, nervous, urinary, respiratory, female genital organs, eye ear and month, hemic and lymphatic, male genital organs, and miscellaneous

^b^Postoperative medications were not included as candidates for covariates or confounding variables of the regression models as those variables could be affected by whether buprenorphine was continued or discontinued postoperatively

For both respiratory depression and respiratory complications outcomes, adjusted odds ratio estimates for the association between buprenorphine continuation and the outcome, with 95% simultaneous confidence intervals, were extracted from the final model using the multcomp package (generalized linear hypotheses function) in R program [14].

R statistical software, version 4.0.2 (R Foundation for Statistical Computing, Vienna, Austria), was used for all statistical analyses. The nominal type I error rate for hypothesis testing procedures and confidence intervals was fixed at 5%.

We anticipated 25% incidence of postoperative respiratory depression in our population based on the preliminary data search performed during the planning stage of the study. This confirmed that our sample size would provide power of 80% to detect a 15% difference in the incidence of respiratory depression between patients who continued versus discontinued buprenorphine postoperatively based on a *Z* test for independent proportions.

## Results

### Demographic, intraoperative, and postoperative characteristics

**Fig. 1:**
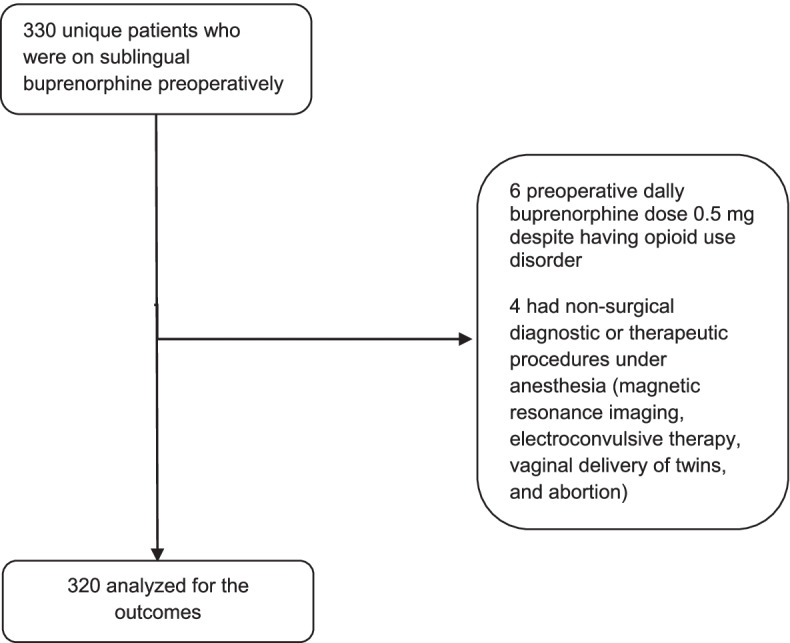
Flow diagram

We retrieved the data of 330 unique patients who were on sublingual buprenorphine preoperatively. In six patients, preoperative daily buprenorphine dose was 0.5 mg despite having opioid use disorder. They were considered to be not on a stable dose of buprenorphine, rather in the process of uptitration of buprenorphine. Four patients had non-surgical diagnostic or therapeutic procedures under anesthesia. After removal of those patients, data of 320 patients were subjected to analysis (Fig. 1). Preoperative daily dose of buprenorphine was not significantly different between the patients who continued versus discontinued buprenorphine postoperatively (*P*=0.06), but a higher proportion of patients who continued buprenorphine postoperatively took the medication for opioid use disorder as opposed to chronic pain (*P*=0.01). Over the study period (2013 to 2020), the practice has shifted from discontinuing buprenorphine to continuing buprenorphine postoperatively (*P*=0.001). There was a significant difference in the makeup of surgery category based on body system between those who continued versus discontinued buprenorphine (*P*=0.02), and higher percentage of patients who continued buprenorphine was on antidepressants preoperatively (*P*=0.02) (Table 1).

### Postoperative opioid dose requirements and pain intensity

Opioid dose requirements in MME for 48 h postoperatively, excluding the dose of buprenorphine, was higher (*P*<0.001), and TWA pain score was higher, in patients who discontinued buprenorphine postoperatively than those who continued, in univariable analysis (*P*<0.001) (Table 2). The patients who continued received 28.0 [12.0 to 40.0] mg of buprenorphine for 48 h postoperatively.

**Table 2:** Opioid dose requirements, buprenorphine dose, and time-weighted average pain scores for 48 h postoperatively. Summary statistics presented as *N* (%) of patients, mean ± SD, or median [IQR]

	Buprenorphine continued (*n*=121)	Buprenorphine discontinued (*n*=199)	*P* value
Postoperative opioid dose in MME, excluding buprenorphine	88 [20–157]	194 [69–343]	< 0.001
Postoperative buprenorphine dose in mg	28.0 [12.0–40.0]	N/A	N/A
Time-weighted average pain score	5.3 ± 2.4	6.2 ± 1.9	<0.001
Length of hospital stay in days	6 [4–10]	6 [4–13]	0.31
Respiratory depression, *N* (%)	33 (27.3)	46 (23.1)	0.40
Respiratory rate < 10/minute	11 (9.1)	15 (7.5)	0.62
SpO_2_ < 90%	26 (21.5)	36 (18.1)	0.46
Requirement of naloxone	0 (0)	0 (0)	-
Respiratory complications, *N* (%)	13 (10.7)	38 (19.1)	0.048
Pulmonary insufficiency following trauma and surgery	0 (0)	1 (0.5)	
Other pulmonary insufficiency, not elsewhere classified	0 (0)	0 (0)	
Acute respiratory failure	3 (2.5)	13 (6.5)	
Other respiratory anomalies	0 (0)	0 (0)	
Pulmonary collapse	1 (0.8)	5 (2.5)	
Pneumococcal pneumonia	0 (0)	0 (0)	
Other bacterial pneumonia	0 (0)	0 (0)	
Bronchopneumonia, organism unspecified	0 (0)	0 (0)	
Pneumonia, organism uspecified	1 (0.8)	1 (0.5)	
Respiratory arrest	0 (0)	0 (0)	
Apnea	0 (0)	0 (0)	
Acute respiratory distress syndrome	2 (1.7)	1 (0.5)	
Respiratory failure, unspecified, unspecified whether with hypoxia or hypercapnia	1 (0.8)	5 (2.5)	
Atelectasis	8 (6.6)	21 (10.6)	
Other pulmonary collapse	2 (1.7)	2 (1.0)	
Pneumonia due to Streptococcus pneumoniae	0 (0)	0 (0)	
Pneumonia due to Hemophilus influenzae	0 (0)	0 (0)	
Bacterial pneumonia, not elsewhere classified	1 (0.8)	0 (0)	
Pneumonia, unspecified organism	8 (6.6)	11 (5.5)	
Apnea, not elsewhere classified	0 (0)	0 (0)	

*Abbreviations*: *SD* standard deviation, *IQR* interquartile range, *MME* morphine milligram equivalents, *N/A* not applicable

### Univariable association between postoperative buprenorphine continuation and respiratory depression and respiratory complications

The incidence of respiratory depression for 48 h postoperatively was not significantly different between those who continued versus discontinued buprenorphine, at 27.3% and 23.1%, respectively (*P*=0.40). Incidences of components of respiratory depression (i.e., respiratory rate < 10/minute and SpO_2_ < 90%) were not different between the two groups, and no patients required naloxone (Table 2). The detail of the components of the composite of respiratory complications is described in Supplemental Table 1. Respiratory complications were less frequent in those who continued buprenorphine than discontinued, with the incidences 10.7% and 19.1%, respectively (*P*=0.048). The three most common diagnosis of respiratory complications were atelectasis (ICD-10: J98.11), pneumonia, unspecified organism (ICD-10: J18), and acute respiratory failure (ICD-10: J96.0) (Table 2).

In the subgroup of preoperative low-dose buprenorphine (<16 mg daily), a continuation was not significantly associated with the incidence of respiratory depression (unadjusted odds ratio (OR), 0.71, 95% confidence interval (CI), 0.35 to 1.42). In the high-dose subgroup (≥16 mg daily), continuation was associated with a higher incidence of respiratory depression (unadjusted OR, 3.23, 95% CI, 1.36 to 7.69).

In the low-dose subgroup (<16 mg daily), continuation was associated with a lower incidence of respiratory complications (unadjusted OR, 0.32, 95% CI, 0.12 to 0.88). In the high-dose subgroup (≥16 mg daily), continuation was not associated with the incidence of respiratory complications (unadjusted OR, 0.84, 95% CI, 0.32 to 2.19).

### Multivariable association between postoperative buprenorphine continuation and postoperative respiratory depression

The multivariable model showed good discrimination (a c-statistic equal to 0.71) and calibration (a Hosmer-Lemeshow Goodness-of-Fit test with *P*=0.78). Postoperative buprenorphine continuation was not associated with postoperative respiratory depression (adjusted OR, 1.11, 95% CI, 0.61 to 1.99, *P*=0.72).

The results of subanalysis stratified by preoperative buprenorphine dose are summarized in Table 3. The model term representing the hypothesized interaction between postoperative buprenorphine continuation and preoperative buprenorphine dose was not statistically significant (likelihood-ratio test: *P*=0.056) (Table 3), but the evidence was marginal (i.e., *P* value near 0.05). Further, in univariable analysis, buprenorphine continuation demonstrated non-statistical trend of protection against postoperative respiratory depression when preoperative dose was low (i.e., unadjusted OR, 0.71 for low dose), while buprenorphine continuation increased the risk of postoperative respiratory depression with statistical significance when preoperative dose was high (i.e., unadjusted OR, 3.23 for high dose). Therefore, postoperative buprenorphine continuation x preoperative buprenorphine dose interaction term was included in the multivariable model of the subanalysis. When preoperative buprenorphine dose was low (<16 mg daily), continuation was not associated with postoperative respiratory depression (adjusted OR, 0.71, 95% CI, 0.30 to 1.68, *P*=0.599). When preoperative buprenorphine dose was high (≥16 mg daily), continuation was not associated with postoperative respiratory depression (adjusted OR, 2.19, 95% CI, 0.76 to 6.27, *P*=0.182) (Table 4).

**Table 3:** Summary of multivariable regression model for respiratory depression

Term	Estimated coefficient	95% CI lower	95% CI upper	Estimated odds ratio	*P* value
Intercept	−3.06	−4.57	−1.63	0.05	-
Postoperative buprenorphine continuation (yes)	−0.35	−1.12	0.39	-	0.367
Year of surgery	0.18	0.0	0.36	1.20	0.054
Facility (UWMC)	0.22	−0.39	0.82	1.25	0.468
Regional anesthesia (yes)	−0.73	−1.63	0.05	0.48	0.082
BMI, kg/m^2^	0.05	0.01	0.09	1.05	0.011
Preoperative antidepressant (yes)	0.73	0.17	1.30	2.08	0.010
Preoperative buprenorphine dose (≥ 16mg)	−0.92	−1.76	−0.14	-	0.025
Emergency status (yes)	−0.96	−1.77	−0.23	0.38	0.014
Postoperative buprenorphine continuation* Preoperative buprenorphine dose ≥16mg	1.13	−0.02	2.32	-	0.058

Hosmer-Lemeshow GOF *X*^2^ = 9.5 (8 df), *p*=0.3, *c*=0.72, LRT for interaction term: *p*=0.056

*Abbreviations*: *UWMC* University of Washington Medical Center, *BMI* body mass index, *GOF* goodness of fit, *LRT* likelihood ratio test

**Table 4:** Adjusted odds ratio estimates and 95% confidence intervals for respiratory depression for patients who continued buprenorphine postoperatively, based on multivariable regression model

Preoperative buprenorphine dose	Odds ratio estimate	95% CI lower	95% CI upper	*P* value
< 16mg daily	0.71	0.30	1.68	0.599
≥16mg daily	2.19	0.76	6.27	0.182

*Abbreviations*: *CI* confidence interval

### Multivariable association between postoperative buprenorphine continuation and respiratory complications

The results of the multivariable logistic regression model for postoperative respiratory complications are summarized in Table 5. This multivariable model showed good discrimination (a c-statistic of 0.69) and calibration (a Hosmer-Lemeshow Goodness-of-Fit test of *P*=0.99). The model term representing the hypothesized interaction between postoperative buprenorphine continuation and preoperative buprenorphine dose was not statistically significant (likelihood-ratio test: *P*=0.13), and the univariable association between buprenorphine continuation and postoperative respiratory complications was in the same direction when preoperative buprenorphine dose was low and high (i.e., unadjusted OR, 0.32 for low dose, and unadjusted OR, 0.84 for high dose). Therefore, postoperative buprenorphine continuation x preoperative buprenorphine dose interaction term was not included in the multivariable regression model for this outcome. Postoperative buprenorphine continuation was associated with lower incidence of respiratory complications (adjusted OR, 0.43, 95% CI, 0.21 to 0.86, *P*=0.02) (Table 5).

**Table 5:** Summary of multivariable regression model for respiratory complications

Term	Estimated coefficient	95% CI lower	95% CI upper	Estimated odds ratio	*P* value
Intercept	−1.33	−2.70	0.02	0.26	-
Postoperative buprenorphine continuation (yes vs. no)	−0.83	−1.60	−0.15	0.43	0.02
Surgery category					
Musculoskeletal (vs. digestive)	−1.19	−2.04	−0.31	0.31	0.007
Integumental (vs. digestive)	−0.87	−2.00	0.19	0.42	0.11
Other (vs. digestive)	−0.37	−1.29	0.55	0.69	0.42
Regional anesthesia (yes vs. no)	−0.50	−1.54	0.39	0.61	0.31
BMI, kg/m^2^	0.03	−0.01	0.07	1.03	0.11
Indication for buprenorphine (Chronic pain vs. opioid use disorder)	−0.45	−1.28	0.30	0.64	0.26
Preoperative buprenorphine dose (≥16mg vs. < 16 mg)	−0.10	−0.76	0.55	0.90	0.76

Hosmer-Lemeshow GOF *X*^2^ = 9.5 (8 df), *P*=0.99, *c*=0.69, LRT for interaction term: *P*=0.13

*Abbreviations*: *CI* confidence interval, *BMI* body mass index, *GOF* goodness of fit, *LRT* likelihood ratio test.

## Discussion

Among inpatient surgical patients taking sublingual buprenorphine preoperatively, continuation during the first 48 h postoperatively was not associated with postoperative respiratory depression. However, postoperative buprenorphine continuation was associated with 57% decreased odds of respiratory complications than postoperative discontinuation. As postoperative respiratory complications have major impact on morbidity and also leads to increased length of hospital stay, continuation of buprenorphine might lead to significant cost saving.

When stratified by preoperative buprenorphine dose, postoperative buprenorphine continuation was associated with non-statistically significant trend of decreased respiratory depression when preoperative buprenorphine dose was low (< 16 mg daily) and with non-statistically significant trend of increased respiratory depression when preoperative buprenorphine dose was high (≥16 mg daily).

Opioid dose requirements (excluding buprenorphine dose given postoperatively) and time weighted average pain scores during postoperative 48 h were lower when buprenorphine was continued postoperatively. This may be partially explained by the analgesic effect of buprenorphine. As extensive dose-response relationship of the analgesic effect of buprenorphine over the entire clinical dose range has not been available, the dose at which buprenorphine reaches analgesic ceiling effect as a partial mu-agonist is not known. However, at least in the range of daily buprenorphine dose given postoperatively in our study (i.e., 14.0 [6.0–20.0] (median [Q1–Q3])), buprenorphine seemed to exert additive analgesic effect with other mu-agonists.

The most frequently reported components of composite of respiratory complications were atelectasis, pneumonia, and acute respiratory failure. Therefore, the lower incidence of respiratory complications when buprenorphine was continued versus discontinued are mainly attributed to the difference in the incidences of those components. Lower TWA pain score with continuation (5.3 ± 2.4, versus 6.2 ± 1.9 with discontinuation) could at least partially explain the association between continuation and the lower incidence of respiratory complications. Half of the patients included in the current study underwent surgeries involving musculoskeletal system, and associations between lower incidences of pneumonia and respiratory failure and lower amounts of opioid consumption have been reported after total knee arthroplasty [15]. Therefore, it seems that pain, and immobility caused by pain, increase the risk of postoperative respiratory complications in those undergoing musculoskeletal surgery.

There are several limitations inherent to the current study. We used respiratory rate < 10/minute, SpO_2_ < 90%, and requirement of naloxone as the definition of respiratory depression (none of our patients received naloxone) in line with the cutoff values for respiratory rate and SpO_2_ described in the practice guidelines for the American Society of Anesthesiologists (ASA) as clinical signs of opioid-induced respiratory depression [16]. Respiratory rate and SpO_2_ were collected through routine checks every 4 h by ward nurses in the majority of our patients. In a study of continuous oxygen saturation monitoring for 48 h following non-cardiac surgery, 90% of serious hypoxemic events (SpO_2_ < 90% for ≥ 1 h) were missed by routine every 4 h vital sign checks [17]. Therefore, it is very likely that many hypoxemic events were undetected and the true incidence of hypoxemia was much higher in both patients who continued and discontinued buprenorphine postoperatively.

Another limitation is the use of ICD-9 and ICD-10 codes to capture postoperative respiratory complications. Although the incidence of postoperative complications based on ICD coding, and that based on prospective assessment of complications, have been reported to be similar, ICD coding captured events of minor clinical importance [18]. Therefore, the incidence rates of respiratory complications in the current study might be somewhat inflated.

## Conclusions

Continuation of buprenorphine for the first 48 h postoperatively was not associated with postoperative respiratory depression. Postoperative buprenorphine continuation was associated with a lower incidence of postoperative respiratory complications than postoperative discontinuation.

## Supplementary Information

**Additional file 1: Table S1**. Description of individual components of postoperative respiratory complications.

## Data Availability

The data that support the finding of this study are available from the corresponding author, RK, upon reasonable request.

## Abbreviations

SpO_2_: Oxygen saturation
OR: Odds ratio
CI: Confidence interval
IRB: Institutional review board
ID: Identification number
UWMC: University of Washington Medical Center
HMC: Harborview Medical Center
STROBE: Strengthening the Reporting of Observational Studies in Epidemiology
AHRQ: Agency for Healthcare Research and Quality
RVU: Relative value unit
CPT: Current procedural terminology
ICD: International Classification of Diseases
TWA: Time weighted average
MME: Morphine milligram equivalents
NRS: Numeric rating scale
ASA: American Society of Anesthesiologists
