# Supplementary material for: Postoperative respiratory depression in patients on sublingual buprenorphine: a retrospective cohort study for comparison between postoperative continuation and discontinuation of buprenorphine
Source: JA Clin Rep. 2022 Jun 21;8:45. doi: 10.1186/s40981-022-00535-2 (PMC9209540; doi:10.1186/s40981-022-00535-2)
Supplement: Supplementary file 1 — Additional file 1: Table S1. Description of individual components of postoperative respiratory complications. [file 40981_2022_535_MOESM1_ESM.docx]

| **Supplemental Table 1.** Description of individual components of postoperative respiratory complications | | |
| --- | --- | --- |
| **ICD-code** | **Description** |  |
|  |  |  |
| **ICD-9** |  |  |
| 481 | Pneumococcal pneumonia |  |
| 482 | Other bacterial pneumonia |  |
| 485 | Bronchopneumonia, organism unspecified |  |
| 486 | Pneumonia, organism uspecified |  |
| 518.0 | Pulmonary collapse |  |
| 518.5 | Pulmonary insufficiency following trauma and surgery |  |
| 518.81 | Acute respiratory failure |  |
| 518.82 | Other pulmonary insufficiency, not elsewhere classified |  |
| 786.03 | Apnea |  |
| 786.09 | Other respiratory anomalies |  |
| 799.1 | Respiratory arrest |  |
| **ICD-10** |  |  |
| J13 | Pneumonia due to Streptococcus pneumoniae |  |
| J14 | Pneumonia due to Hemophilus influenzae |  |
| J15 | Bacterial pneumonia, not elsewhere classified |  |
| J18 | Pneumonia, unspecified organism |  |
| J80 | Acute respiratory distress syndrome |  |
| J96.0 | Acute respiratory failure |  |
| J96.90 | Respiratory failure, unspecified, unspecified whether with hypoxia or hypercapnia |  |
| J98.11 | Atelectasis |  |
| J98.19 | Other pulmonary collapse |  |
| R06.81 | Apnea, not elsewhere classified |  |
| R09.2 | Respiratory arrest |  |
| Abbreviations: ICD-9 = International Classification of Diseases, ninth revision**,** ICD-10 = International Classification of Diseases, tenth revision | | |
